# Carnitine Palmitoyltransferase 1b Deficiency Protects Mice from Diet-Induced Insulin Resistance

**DOI:** 10.4172/2155-6156.1000361

**Published:** 2014-04-25

**Authors:** Teayoun Kim, Lan He, Maria S. Johnson, Yan Li, Ling Zeng, Yishu Ding, Qinqiang Long, John F. Moore, Jon D. Sharer, Tim R. Nagy, Martin E. Young, Philip A. Wood, Qinglin Yang

**Affiliations:** 1 Department of Nutrition Sciences, University of Alabama at Birmingham, USA; 2 Department of Anatomy, Guangzhou University of Chinese Medicine, Higher Education Mega Center Campus, China; 3 Department of Genetics, University of Alabama at Birmingham, USA; 4 Department of Medicine, University of Alabama at Birmingham, USA; 5 Sanford-Burnham Medical Research Institute at Lake Nona, Orlando, USA

**Keywords:** CPT1b, Fatty acid oxidation, Insulin sensitivity, Skeletal muscle

## Abstract

**Background:**

Carnitine Palmitoyl Transferase 1 (CPT1) is the rate-limiting enzyme governing long-chain fatty acid entry into mitochondria. CPT1 inhibitors have been developed and exhibited beneficial effects against type II diabetes in short-term preclinical animal studies. However, the long-term effects of treatment remain unclear and potential non-specific effects of these CPT1 inhibitors hamper in-depth understanding of the potential molecular mechanisms involved.

**Methods:**

We investigated the effects of restricting the activity of the muscle isoform CPT1b in mice using heterozygous CPT1b deficient (*Cpt1b*^+/−^) and Wild Type (WT) mice fed with a High Fat Diet (HFD) for 22 weeks. Insulin sensitivity was assessed using Glucose Tolerance Test (GTT), insulin tolerance test and hyperinsulinemic euglycemic clamps. We also examined body weight/composition, tissue and systemic metabolism/energetic status, lipid profile, transcript analysis, and changes in insulin signaling pathways.

**Results:**

We found that *Cpt1b*^+/−^ mice were protected from HFD-induced insulin resistance compared to WT littermates. *Cpt1b*^+/−^ mice exhibited elevated whole body glucose disposal rate and skeletal muscle glucose uptake. Furthermore, *Cpt1b*^+/−^ skeletal muscle showed diminished *ex vivo* palmitate oxidative capacity by ~40% and augmented glucose oxidation capacity by ~50% without overt change in whole body energy metabolism. HFD feeding *Cpt1b*^+/−^ but not WT mice exhibited well-maintained insulin signaling in skeletal muscle, heart, and liver.

**Conclusion:**

The present study on a genetic model of CPT1b restriction supports the concept that partial CPT1b inhibition is a potential therapeutic strategy.

## Introduction

Increased fatty acid availability and the concomitantly augmented mitochondrial fatty acid oxidation (FAO) capacity in skeletal muscle is a common feature in insulin resistant subjects [1,2]. Interfering with excessive fatty acid availability and oxidation hence may be a potential therapeutic strategy. Accumulated evidence has emerged that inhibiting FAO may sensitize insulin signaling in animals with insulin resistance [3–7] and in diabetic patients [7–9]. Carnitine Palmitoyl Transferase 1 (CPT1) on the mitochondrial outer membrane is a rate limiting enzyme that converts long-chain acyl CoA into long chain acylcarnitine, thus being one of the most studied therapeutic targets for FAO inhibition.

There are three isoforms of CPT1 (a, b and c). While CPT1a is expressed ubiquitously but most abundantly in the liver, CPT1b is expressed in skeletal muscle, heart, and adipose tissues, and CPT1c is expressed in the brain and testes (reviewed in [10]). HFD-fed mice show increased capacity of fatty acid oxidation in skeletal muscle homogenate and mitochondrial respiration with concomitantly increased CPT1 activity [11]. The expression of CPT1bis inversely related to insulin sensitivity among quartiles of metabolic syndrome patients [12]. Repressing the capacity of fatty acid oxidation may ameliorate insulin resistance. In supporting this hypothesis, several groups reported that CPT1 inhibitors improve insulin resistance in relatively short-term preclinical animal studies [5–8,13,14], even with increased intracellular lipid content. CPT1 inhibition in molecular genetic models due to the deletion of Malonyl-CoA-Decarboxylase (MCD) shows similar effects. The MCD null (*Mcd*^−^*^/^*^−^) mice are protected from HFD-induced insulin resistance by substantially increased Malonyl-CoA-Mediated repression of CPT1, consequently decreasing fatty acid oxidation and increasing glucose oxidation [3]. On the other hand, a 20% electroporation-mediated transient upregulation of CPT1b in the distal hind limb muscles is sufficient to improve HFD-induced insulin resistance in rats via attenuating triacylglycerol content, the membrane-to-cytosolic ratio of diacylglycerol, and protein kinase Cθactivation [15]. Therefore, further studies on the *in vivo* roles of specific CPT1 isoform using the more specific gene manipulating approach are essential to resolve these seemingly contradictory observations. The goal of the present study is to elucidate the effects of restricting fatty acid oxidation on diet-induced insulin resistancein the heterozygous *Cpt1b* knockout mice. This is the first study using a preclinical mouse model with *Cpt1b*-specific knockdown to investigate the long-term effects of CPT1b repression on HFD-induced insulin resistance and the underlying metabolic mechanisms.

## Materials and Methods

### Animals

The heterozygous *Cpt1b*^+/−^ knockout mice [16] and wild-type (WT) littermates in C57BL/6J background were used. While the homozygous CPT1b knockout is lethal, *Cpt1b*^+/−^ mice show no overt abnormal phenotype and whole body metabolic changes [16]. All mice were kept on 12-hour/12-hour light/dark cycle (light on at 06:00 hours) at 22.0 ±1.0°C and had *ad libitum* access to water and standard rodent diet (Harlan Laboratories 7017 NIH-31 Mouse/Rat Sterilizable Diet, 14% kcal% fat). Mice (4 – 5 week old, male) of high fat diet (HFD) feeding groups were given *ad libitum*access to HFD (60% kcal% fat) (Research DIETS D12492) and water. Body weights were recorded every other week. All experimental procedures were conducted in accordance with the Guide for Care and Use of Laboratory Animals and were approved by the Institutional Animal Care and Use Committee of the University of Alabama at Birmingham (UAB).

### Oral Glucose Tolerance Test (OGTT) and Insulin Tolerance Test (ITT)

Mice were fasted overnight for OGTT (18:00 – 08:00) or 5 hours for ITT (08:00–13:00). Blood glucose levels were measured on blood samples from tail tip-snip using Contour glucometer (Bayer) after glucose administration (gavage 1.5 mg/kg b.w.) or insulin injection (*Humulin*^®^ *R,* Eli Lilly & Co, i.p. 0.5 U/kg b.w.).

### Hyperinsulinemiceuglycemic clamp

The procedures of hyperinsulinemiceuglycemic clamp (insulin clamp) in mice were adapted from Dr. Pessin’s group[17] with minor modifications based on reports from other groups [18,19]. Briefly, mice were anesthetized by Isoflurane via a Vaporizer-MiniVentmouse ventilator system (HUGO SACHS ELECTRONIK, Harvard Apparatus GmbH, Hugstetten, Germany). A catheter was surgically implanted into the right jugular vein and threaded under the dorsal skin of mice. Three days after surgery, the mouse was fasted 5 hrs (08:00–13:00) and then placed in a rat-size restrainer with its tail taped. The catheter was connected to a CMA 402 syringe pump (CMA Microdialysis, Stockholm, Sweden). [6-^3^H]-glucose was infused at 0.5 μCi/min for 2 hrs without insulin and then infused at 1μCi/min with insulin (Humulin R, Eli Lilly 2.5 mU kg^−1^ min^−1^) for 2 hrs, by which time the blood glucose was maintained at 7.8 – 8.9 mmol/L by adjusting 20% glucose infusion rate in the mouse under the conscious condition. 10 μCi 2-[^14^C]-deoxy-D-glucose was infused 40 min before the end of the 120 min euglycemic clamp. The blood glucose level was measured from tail tip snipped blood samples using a Contour glucometer (Bayer). At the end of the clamp study, tissues were harvested and snapped frozen in liquid nitrogen after the mouse was euthanized. The plasma glucose level was measured using an Analox GM7 Micro-Stat Analyzer (Analox Instruments, London, UK). The specific activity of plasma glucose, the glucose infusion rate (GIR), the whole body glucose disposal rate (Gd), and the tissue-specific glucose uptake were measured and calculated as previously described [20].

### Serum analysis

Tissues and sera were collected from sacrificed mice after overnight fasting (18:00 – 08:00). Insulin level was measured using a RIA kits (Millipore Co. SRI-13K, ML-82K). The content of Non-Esterified Fatty Acids (NEFA) was measured using a NEFA-HR Kit (Wako), respectively.

### Lipid measurements

Frozen gastrocnemius muscles were pulverized using a pulverizor (Bio Spec Products Inc.) in liquid nitrogen and weighed in small tubes as previously described [21]. For the non-esterified fatty acids (NEFA), lipids were extracted using the Bligh & Dyer method [22]. NEFA and TAG were measured using a NEFA-HR Kit (Wako) and a Triglyceride Quantification Kit (BioVision K622-100). For the acylcarnitine assay, 6 volume of 80 % acetonitrile was added to pulverized tissue weight (about 50 mg). Tissue mixtures were sonicated 10 times, centrifuged at 12,000 rpm 10 min at 4 °C, and supernatants were transferred to new tubes. The supernatants were dried under a stream of nitrogen at 40 °C and resuspended in 100 μl of 50% acetonitrile. The acylcarnitine content was measured by using Electrospray Ionization Tandem Mass Spectrometry [23].

### *Ex vivo* oxidation assay

Intact muscle oxidation assay was performed as previously described [24]. Extensor digitorumlongus (EDL) muscles were excised from euthanized mice and incubated with 700 μl of Krebs-Ringer Phosphate buffer containing 0.1 μCi/ml of BSA-conjugated [^14^C]-palmitateor [^14^C]-glucose in sealed 14 ml tubes with center wells containing 1N NaOH at 37°C for 1 hour with 200 rpm shaking. After incubation, 400 μl of 3.5 M HClO_4_ was injected into the media and incubated at 50°C for 3 hours to capture oxidized substrates to NaOH and the radioactivity was measured by scintillation counter [24].

### Oxidation assays in isolated mitochondria

The procedures of mitochondrial oxidation assay were adapted from Dr. Kove’s group as previously described [3]. Gastrocnemius muscle (one hind limb from one mouse) was homogenized by using hand-held drill with Potter-Elvehjem homogenizer. Supernatants were kept following a low speed (1,000 xg) centrifugation, and subsequently centrifuged again at 12,000 xg to isolate mitochondria. Mitochondrial protein concentration was measured by Lowry method and 100 μg of mitochondria was incubated with 0.1 μCi/ml of [^14^C]-palmitate including reaction buffer for 2 hrs. The oxidation reaction was stopped by injecting perchloric acid. Oxidized substrates were metabolized as ^14^CO_2_ and captured by NaOH in the central well and the radioactivity was measured by scintillation counter. Incompletely oxidized substrates (acid soluble metabolites-ASM) remained in the reaction mixture were centrifuged. Then the supernatants were used to detect ^14^C by liquid scintillation counter.

### Real-time qPCR

Total RNA extraction and cDNA synthesis were done by using RNeasy^®^ Mini kit (QIAGEN), Advantage^®^ RT-for PCR kit (Clontech), *Power* SYBR^®^ Green PCR master mix. The real-time PCR was performed by using Step One Real-Time PCR System (Applied Biosystems) with standard curve for the transcript quantification. The primer sequences are listed in Supplementary Table 1.

### Western blot analysis

Frozen gastrocnemius muscles, liver, and hearts were homogenized using a pestle pellet mini homogenizer in homogenization buffer (50 mM TrisHCl pH 6.8, 1% SDS, 2.5 mM DTT, 10% glycerol). The protein concentration of supernatant was measured by using Modified Lowry Protein Assay Kit (Pierce #23240). Primary antibodies were purchased from Cell Signaling; pIRS1 Ser302 (#2491), IRS1 (#2390), pAKT Ser473 (#9271), AKT (#9272), pERK1/2 (p44/42 MAPK) Thr202/Tyr204 (#9101), and ERK1/2 (#9102). HRP-conjugated secondary antibodies were from Santa Cruz Biotechnology. Western blot images were taken and quantified by using Kodak Image Station 4000R (Molecular Imaging System, Carestream Health Inc., Rochester, NY, USA).

### Body composition analysis

Fat and lean mass were measured *in vivo* using a quantitative magnetic resonance imaging system (QMR, EchoMRI^™^ 3-in-1, Echo Medical System, Houston, TX, USA) at UAB Small Animal Physiology Core as previously described and validated [25].

### Comprehensive Lab Animal Monitoring System (CLAMS) analysis

Respiration rate, food intake, energy expenditure, and physical activity were accurately quantified as previously described using the CLAMS (Columbus Instruments Inc., OH, USA) [26]. Mice were individually kept in CLAMS chambers with *ad libitum* access to food and water for 6 days and the data sets for the last 3 days were averaged for the analysis. Respiratory exchange ratio (RER) was calculated as CO_2_ generation/O_2_ consumption. Energy expenditure was expressed as (kcal/hr).

### Indirect calorimetry analysis

Energy expenditure, RER, and activity were also measured with another set of mice using an 8-cage indirect calorimetry system (CaloSys, TSE Systems, Bad Homburg, Germany) as previously described [27]. Mice were individually kept in airtight plastic cages with *ad libitum* access to food and water and a continuous flow of air was maintained through all cages. Mice were acclimated to the cages for 48 hours prior to the measurement period of 22 hours. Total energy expenditure was calculated over the 22 hours, and presented as per 24-hours. Resting energy expenditure was calculated as the average of the 3 lowest 18 minute periods during the measurement.

### Rectal temperature measurement

Body temperatures of mice were measured using rectal probe provided from Echocardiogram (Vevo770, Visualsonics Inc., Toronto, Ontario, Canada) under isoflurane-induced anesthesia at around 13:00.

### Statistical analysis

*GraphPad Prism5* software was used to conduct Two-tailed Student’s *t*-test and One-Way ANOVA with Tukey post hoc test. The ANCOVA was done using *SAS9.1* software. Differences between groups were regarded significant at p<0.05 probability level. Data are expressed as means ± SE.

## Results

### Mice with CPT1b deficiency are protected against HFD-induced insulin resistance

**Figure 1:**
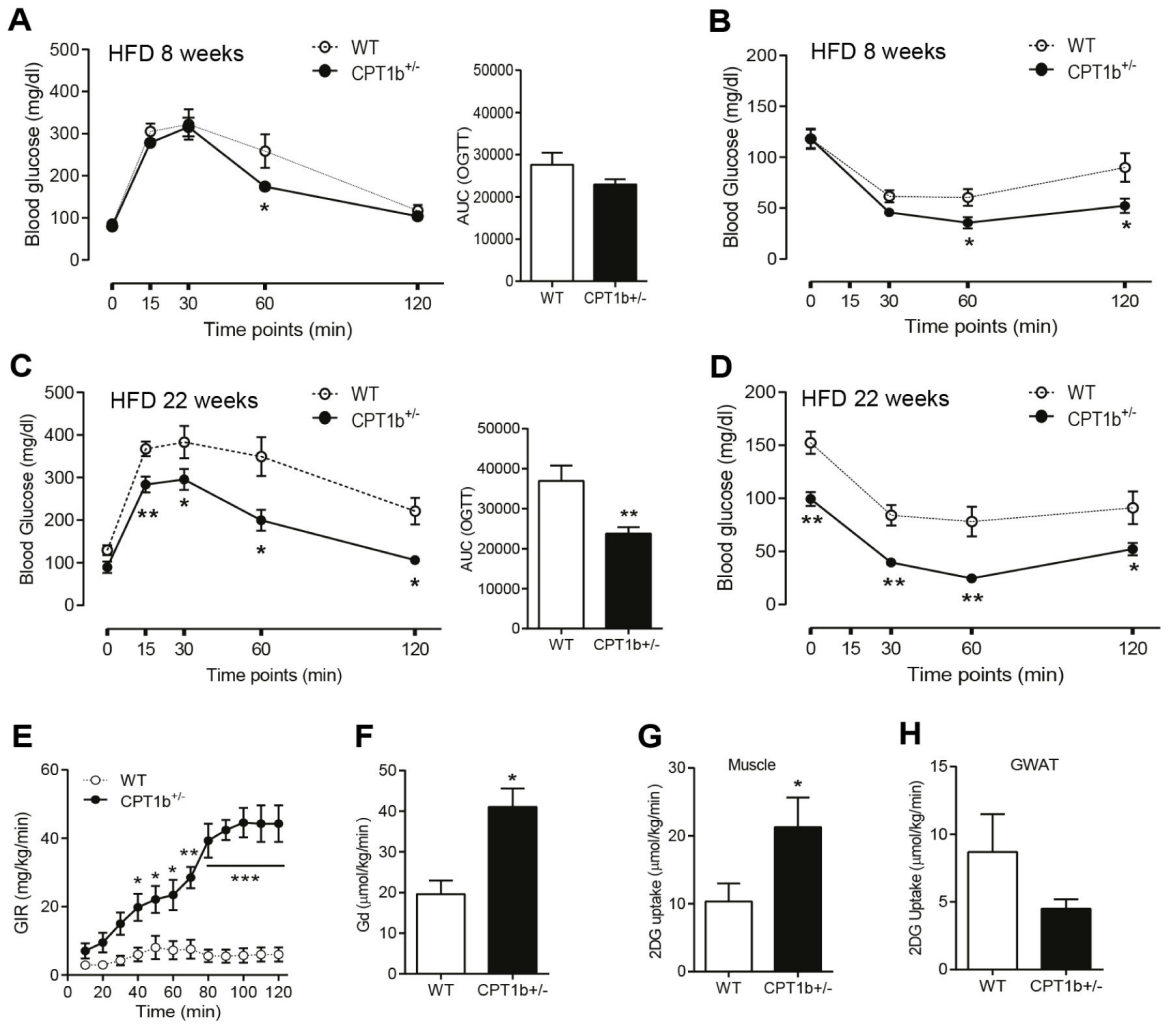
*Cpt1b*^+/−^ mice are insulin sensitive under a HFD condition Insulin sensitivity was assessed by OGTT, ITT, and Insulin clamp study at different time points. (A) OGTT in mice with 8 weeks of HFD. (B) ITT in mice with 8 weeks of HFD. (C) OGTT in mice with 22 weeks of HFD. (D) ITT in mice with 22 weeks of HFD. Bar graphs are the area under curve (AUC) of OGTT calculated from the original graph. (E) Glucose infusion rate (GIR) during last 40 minutes of insulin-stimulated condition in mice with 22 weeks of HFD. (F) Whole body glucose disposal rate (Gd) in mice with 22 weeks of HFD. (G) Glucose uptake into gastrocnemius muscle in mice with 22 weeks of HFD. (H) Glucose uptake into GWAT in mice with 22 weeks of HFD. *n*=4–6, *p<0.05, **p<0.01, ***p<0.001.

We investigated whether partial CPT1b deficiency determines insulin sensitivity in mice under CHD and HFD feeding conditions using OGTT and ITT. At baseline (CHD 4 weeks), CHD-fed *Cpt1b*^+/−^ and WT littermates showed no difference in OGTT and ITT (Figure 1A and 1B). After 8 weeks of HFD feeding, *Cpt1b*^+/−^ mice showed attenuated blood glucose levels at 60 minutes time point during OGTT (Figure 1C). The blood glucose levels during ITT were not changed at the 30 minute time point, but were lower in *Cpt1b*^+/−^ than in WT mice at 60 and 120 minute time points (Figure 1D). At 22 weeks after HFD feeding, *Cpt1b*^+/−^ mice showed substantially improved glucose tolerance compared with WT littermates. The area under curve (AUC) of OGTT in *Cpt1b*^+/−^ mice was decreased about 45 % compared with WT (Figure 1E). The blood glucose levels during ITT were not changed at the 30 minute time point but lower in *Cpt1b*^+/−^ than in WT mice at the later time points (Figure 1F). These results indicate that CPT1b deficiency improves glucose and insulin tolerance under a HFD condition. *Cpt1b*^+/−^ mice showed no difference in ITT and OGTT until 32 weeks of their age (Supplemental Figure 1) suggesting that CPT1b deficiency does not alter insulin sensitivity and glucose tolerance under a normal diet condition.

To gain insights into the tissue-specific insulin sensitivities, we further assessed HFD feeding mice using the hyperinsulinemiceuglycemic clamp at 22 weeks after HFD feeding. A much higher glucose infusion rate (GIR) in *Cpt1b*^+/−^ mice than in WT mice was required to maintain blood glucose levels (7.8 – 8.9 mmol/L) in response to the constant insulin infusion (Figure 1G). Consistently, whole body glucose disposal rate (Gd) was higher in *Cpt1b*^+/−^ than in WT mice (Figure 1H), indicating improved systemic insulin sensitivity in the *Cpt1b*^+/−^mice compared with WT mice. Insulin-stimulated glucose uptake was increased by about 2-fold in *Cpt1b*^+/−^ relative to WT skeletal muscle (Figure 1), whereas there was no difference between two groups in Gonadal White Adipose Tissue (GWAT)(Figure 1H). The plasma glucose level was significantly lower in *Cpt1b*^+/−^ mice than in WT littermates at 8 weeks and 22 weeks of HFD feeding. No changes could be detected in plasma NEFA throughout the observation period in both groups. On the other hand, plasma insulin (p<0.01) and leptin levels surged after 22 weeks of HFD in WT from 8 weeks, while insulin levels in *Cpt1b*^+/−^ mice were substantially lower than their WT controls at 22 weeks. The plasma insulin level during insulin clamp was not different between two groups, while plasma NEFA level was substantially decreased in *Cpt1b*^+/−^ mice (Table 1).

### CPT1b deficiency in mice leads to repressed fatty acid oxidation and enhanced glucose oxidation in the skeletal muscle under a HFD condition

**Figure 2:**
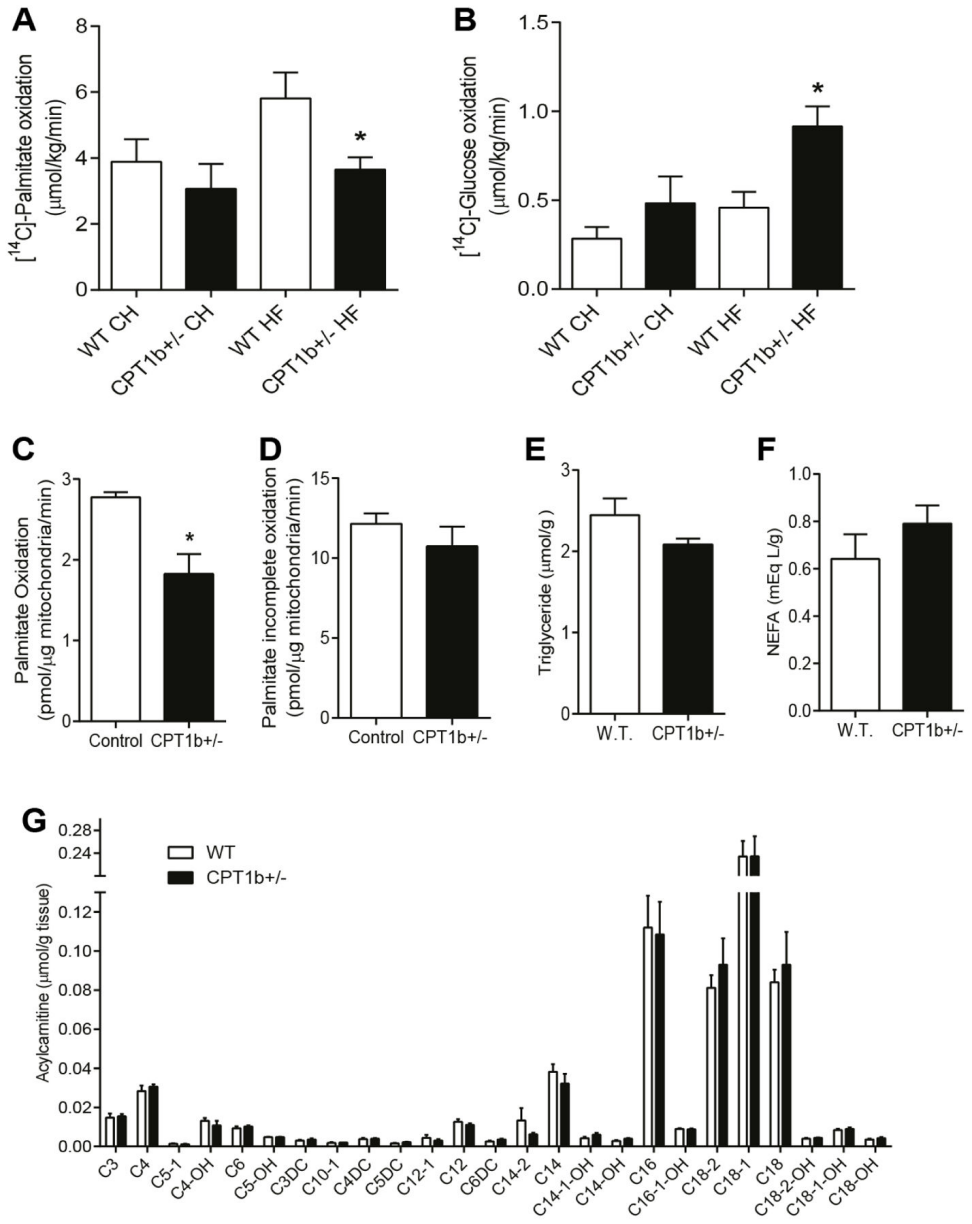
CPT1b deficiency leads to suppressed fatty acid oxidation and increased glucose oxidation without lipid accumulation in the skeletal muscle Substrate oxidation rate and lipid contents in the skeletal muscle of mice were assessed after 22 weeks of HFD feeding. (A) *Ex vivo* [^14^C]-palmitate oxidation in EDL muscle. (B) *Ex vivo* [^14^C]-D-glucose oxidation in EDL muscle. (C) [^14^C]-palmitate complete oxidation in isolated mitochondria from gastrocnemius muscle. (D) [^14^C]-palmitate incomplete oxidation in isolated mitochondria from gastrocnemius muscle. (E) Triglyceride content in gastrocnemius muscle. (F) Non-esterified free fatty acid (NEFA) content in gastrocnemius muscle. (G) Acylcarnitine content in gastrocnemius muscle. *n*=5–6, *p<0.05, **p<0.01, ***p<0.001.

We further investigated whether changes in substrate metabolism correlated with different insulin sensitivity in *Cpt1b*^+/−^ vs. WT mice. CPT1b deficient muscle did not show decreased palmitate oxidation capacity compared to WT muscle under a CHD condition (Figure 2A). The rate of *ex vivo* palmitate oxidation in skeletal muscle of *Cpt1b*^+/−^ mice after 22 weeks of HFD feeding was 40 % lower than in WT mice (p<0.05) (Figure 2A), whereas the rate of *ex vivo* glucose oxidation was 50 % higher in *Cpt1b*^+/−^ than in WT muscles (Figure 2B). Other tissues such as heart, brown adipose tissue, and liver showed no difference in the *ex vivo* oxidation capacity between two groups (data not shown) under a HFD condition. The palmitate oxidation rate in isolated mitochondria was also decreased in CPT1b^+/−^ compared with WT muscle (Figure 2C) with no change in incomplete oxidation (Figure 2D). To assess whether the decreased fatty acid oxidation capacity would result in intramyocellular lipid accumulation, skeletal muscle lipid contents were assessed after 22 weeks of HFD feeding. There was no difference in TAG and NEFA levels between *Cpt1b*^+/−^ and WT muscle (Figure 2E and 2F). Notably, acylcarnitine profile in skeletal muscle of HFD-feeding *Cpt1b*^+/−^ mice was also not different from WT (Figure 2G). In the transcript analysis, *Cpt2* (p<0.01), *Cpt1a, Acs* (acyl-CoA synthase long-chain family member 1), *Dgat* (diacylglycerol O-acyltransferase 2), *Gpam* (glycerol-3-phosphate acyltransferase, mitochondrial), and *Mlycd* (malonyl-CoA decarboxylase) were significantly decreased in *Cpt1b*+/− muscle (P<0.05) (Supplemental Figure 2) supporting that lipid was not accumulated in *Cpt1b*+/− muscle. Therefore, the above results implicate that modest repression of FAO in skeletal muscle could increase glucose oxidation without overt intramuscular lipid accumulation.

### Insulin signal transduction is well maintained in *Cpt1b*^+/−^ mice skeletal muscles

**Figure 3:**
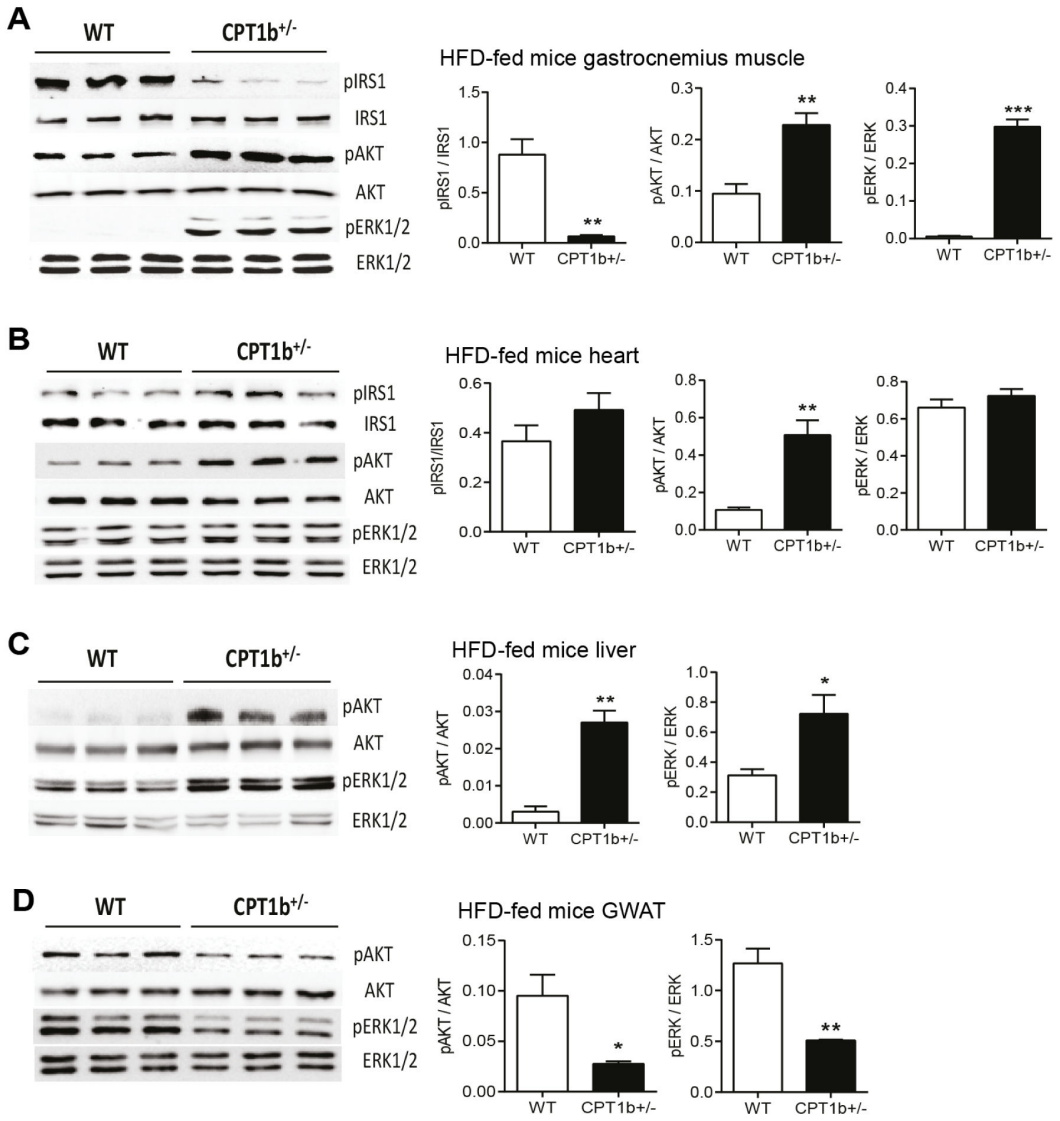
Insulin signaling is well maintained in the skeletal muscle of *Cpt1b*^+/−^ mice Tissue samples from Insulin clamp study after 22 weeks of HFD feeding were subjected to Western blot analysis for the phosphorylation of Ser 302 IRS1, Ser473 AKT, Thr 202/Tyr 204 ERK1/2 and total protein of each target. (A) Gastrocnemius muscle. (B) Heart. (C) Liver. (D) GWAT. *n*=4, *p<0.05, **p<0.01.

To confirm the improved insulin sensitivity of *Cpt1b*^+/−^ mice under a long-term HFD condition on insulin signaling, we subjected tissue samples from hyperinsulinemiceuglycemic clamp study to Western blot analysis of key insulin signaling proteins. The phosphorylation of IRS1 at Ser302 was substantially lower in *Cpt1b*^+/−^ than in WT muscles (p<0.01) (Figure 3A), but there was no difference in the heart (Figure 3B). No signals of phosphorylation of IRS1 at Ser302 were detected in liver and GWAT (data not shown). The phosphorylation of AKT at Ser473 was substantially elevated in muscle (p<0.01) (Figure 3A), heart (p<0.01) (Figure 3B), and liver (p<0.01) (Figure 3C), but decreased in GWAT (Figure 3D) (p<0.05) of *Cpt1b*^+/−^mice. The phosphorylation of ERK1/2 at Thr202/Tyr204 was maintained in muscle (p<0.05) (Figure 3A), liver (p<0.01) (Figure 3C), but had no difference in heart (Figure 3B) and decreased in GWAT (Figure 3D) of *Cpt1b*^+/−^ mice. These results demonstrate that *Cpt1b*^+/−^ mice are protected from HFD-induced impaired insulin signaling in muscle, heart, and liver, but not in GWAT.

### Mice with CPT1b deficiency are smaller than control mice subjected to HFD

**Figure 4:**
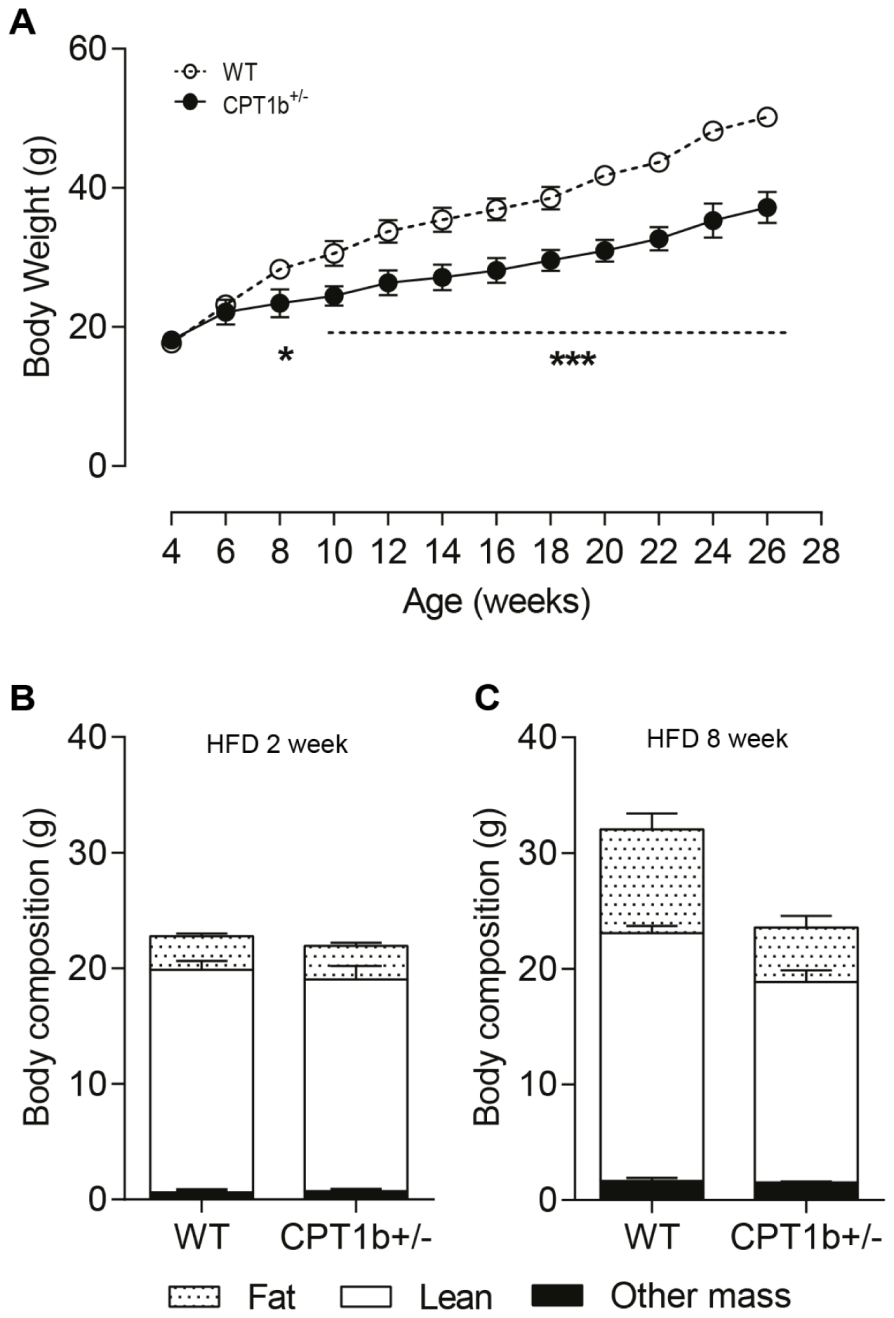
*Cpt1b*^+/−^ mice gain less body weight under a HFD condition (A) Body weight trend in HFD. (B) Body composition (QMR) at 2 weeks of HFD feeding. (C) Body composition (QMR) after 8 weeks of HFD feeding. *n*=6, *p<0.05, ***p<0.001.

We found no difference in body weight and body composition between *Cpt1b*^+/−^ and WT mice under a CHD condition throughout life-time (data not shown). However, *Cpt1b*^+/−^mice showed a smaller increase of body weight than WT mice after 4 weeks of HFD feeding (Figure 4A and 4B). At 8 weeks after HFD feeding, *Cpt1b*^+/−^ mice exhibited 50 % less body fat mass and 14% less lean mass than WT mice (Figure 4C). However, ANCOVA analysis revealed that these changes were proportional to the total body mass, suggesting that the improved insulin sensitivity of *Cpt1b*^+/−^ mice is independent of changes in body fat mass.

### CPT1b deficiency does not influence whole body energy balance in response to HFD feeding

**Figure 5:**
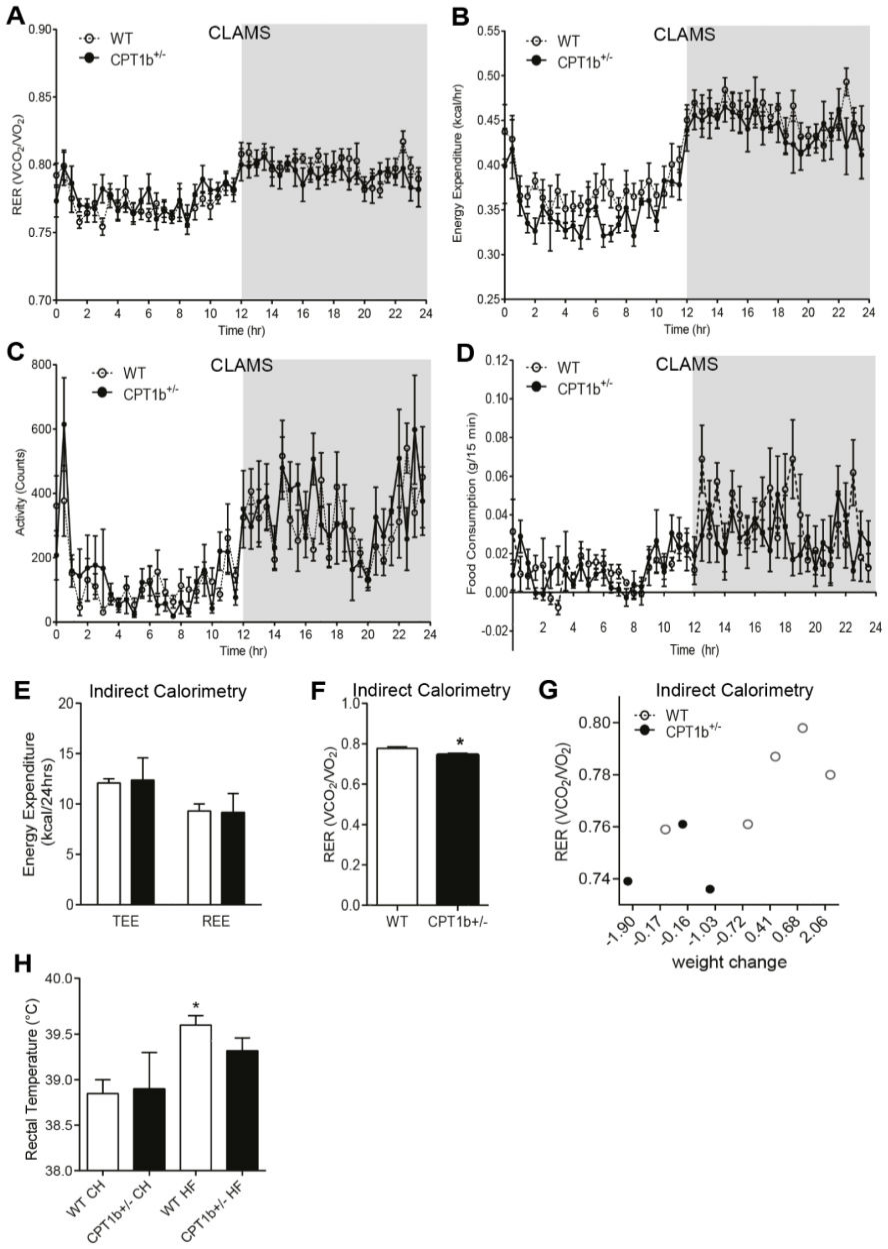
CPT1b deficiency does not alter whole body energy balance under a HFD condition *In vivo* whole body metabolism was assessed at 22 weeks of HFD feeding using comprehensive Lab Animal Monitoring System (CLAMS) with the average of the last 3 days (A–D), *n*=5–6. (A) Respiratory exchange rate (RER). (B) Energy expenditure. (C) Activity. (D) Food intake. Indirect calorimeter analysis (E–G), *n*=3–5. (E) Energy expenditure. (F) Respiratory exchange rate (RER). (G) Correlation between body weights change and RER. (H) Rectal temperature. *n*=4–5, *p<0.05.

We next determined whether the altered substrate oxidation capacity of skeletal muscle in *Cpt1b*^+/−^ mice under a HFD condition affects the whole body energy balance. We measured respiratory exchange rate (RER), energy expenditure, activity, and food intake using CLAMS at 22 weeks after HFD feeding. Interestingly, none of these parameters were different between two groups (Figure 5A–5D). We confirmed the RER assessment of CLAMS analysis by using indirect calorimeter with another set of mice at 22 weeks after HFD feeding. *Cpt1b*^+/−^ mice showed no difference in energy expenditure (Figure 5E) with very subtle decreased RER (Figure 5F), accompanied with weight loss compared to WT during the 2 day measurement period (Figure 5G). Consistently, the HFD-fed WT, but not *Cpt1b*^+/−^ mice, showed higher rectal temperature (Figure 5H), suggesting that the whole body energy metabolism of *Cpt1b*^+/−^ mice was not substantially altered. Analysis of fecal energy content revealed no differences between the two groups (data not shown). Therefore, reduced fatty acid oxidation and increased glucose oxidation in skeletal muscle appear to be in a balanced state that does not substantially alter whole body metabolism and energy balance in mice subjected to HFD.

## Discussion

In the current study, we provide evidence supporting the beneficial effects of CPT1b ablation related fatty acid oxidation restriction against insulin resistance induced by HFD. We investigated a genetic mouse model with *Cpt1b*^+/−^ deficiency subjected to 22 weeks of HFD feeding. We illustrate that CPT1b mediated fatty acid oxidation in skeletal muscle is a crucial site for the maintenance of insulin sensitivity via adjusting the balance of local fatty acid and glucose oxidation rates.

Pharmacological CPT1 inhibition has been suggested as an effective therapy to improve insulin sensitivity in type 2 diabetes patients for nearly two decades [28–30]. A CPT1 inhibitor, etomoxir, improves glucose homeostasis in patients by repressing fatty acid oxidation and elevating glucose oxidation, presumably by the Randle cycle mechanism, despite the subsequent intramyocellular lipid accumulation [7]. Nevertheless, opposite results have also been reported, in which etomoxir is associated with exacerbated insulin resistance with concomitantly increased intracellular lipid accumulation in rodents [31]. Oxfenicine, another CPT1 inhibitor, increases glucose oxidation in HFD-fed mice with increased respiratory exchange ratio (RER), but no intramyocellular lipid accumulation [4]. The inconsistent outcomes from the CPT1 inhibitor studies are most likely derived from the different experimental approaches and the different degrees/subtype specificities of these CPT1 inhibitors. While pharmacological studies provide highly clinical relevant insights into the effects of a specific CPT1 inhibitor, this approach does not target specific CPT1 isoforms with inherent non-CPT1 inhibition effects (e.g., etomoxir has been shown to be a PPARα activator [32]). Moreover, most of previous etomoxir studies were performed with high dosages and relatively short-term assessments on the acute effects of etomoxir, which mostly acts on CPT1a, the liver isoform of CPT1 [33]. Oxfenicine, which inhibits more CPT1b than CPT1a [34], seems to be a promising anti-diabetic drug, since it improves insulin sensitivity without intramuscular lipid accumulation in mice [4]. It remains challenging to exclude the potential off-target effects in pharmacological studies. Additional studies on animal models with genetic manipulations should help overcome the related difficulties. Our current study using the genetic mouse model with CPT1b deficiency provides specific insights and clarifies the effect of CPT1b deficiency on HFD-induced insulin resistance.

Despite the encouraging support of CPT1b inhibition as a potential therapy for insulin resistance as reported here, many other studies using various CPT1 inhibitors have shown adverse effects [31,35] due to intramyocellular fatty acid-related metabolites (e.g., DAGs, acylcarnitines, ceramides) accumulation. In this study we prove that repressed fatty acid oxidation due to heterozygous CPT1b deficiency in skeletal muscle did not lead to accumulation of lipid metabolites, which is consistent with the Oxfenicine study [34]. Previous study showed increased fatty acid oxidation in *ex vivo* muscle tissues of HFD-fed WT mice [11]. Zucker diabetic fatty (ZDF) rats showed increased fatty acid oxidation-related gene expression in their muscle with increased muscle acylcarnitine accumulation [3]. In contrast, a human study showed that palmitate oxidation was significantly decreased, but only in extremely obese patients [36]. Additional studies also demonstrated opposite results either with reduced or elevated fatty acid oxidation in obese human skeletal muscle [37,38]. However, the current study did not detect substantial change at least in the EDL muscle before and after HFD. These different results may be derived from many factors, such as the degrees of obesity and the measurement methods. Decreased palmitate oxidation of the HFD-fed *Mcd*^−/−^ mice improved insulin sensitivity with reduced incomplete fatty acid oxidation [3], supporting the hypothesis that increased incomplete fatty acid oxidation is one of the potential mechanisms of HFD-induced insulin resistance. Unexpectedly, our experiment using the same protocol did not detected any difference of incomplete fatty acid oxidation in isolated mitochondria between *Cpt1b*^+/−^ and WT muscle under a HFD condition. While the reasons for this discrepancy remain incompletely understood, we suspect that blocking the entry of long chain fatty acids into the mitochondria of *Cpt1b*^+/−^ muscle may not be robust enough to cause incomplete fatty acid oxidation, yet is sufficient to repress fatty acid oxidation and upregulating glucose oxidation in skeletal muscle. Therefore, it is probable that partial CPT1b repression protects mice from HFD-induced insulin resistance by attenuating HFD-induced upregulation of fatty acid oxidation and upregulating glucose oxidation in skeletal muscle. Since it has been extensively proposed that intramyocellular lipid accumulation may be one of the main causes of insulin resistance, a potential detrimental effect after prolonged intramyocellular lipid accumulation is still likely. It is possible that the suppressed FAO in *Cpt1b*^+/−^ mice did not reach the threshold that leads to the mismatch of lipid supply and degradation and subsequent intramyocellular lipid accumulation.

The insulin sensitizing effect of CPT1b deficiency is also evident by the remarkable difference in the insulin-stimulated phosphorylation of Ser302 of insulin receptor substrate 1 (IRS1) in the skeletal muscle. Ser302 in rat/mouse IRS-1 (corresponding to Ser307 of human IRS-1) is one of the molecular mechanisms proposed as an indicator of cellular energy status underpinning the development of insulin resistance [39,40]. Transgenic mice with muscle-specific IRS-1 serine to alanine are protected from fat-induced insulin resistance in skeletal muscle [41]. Since the phosphorylation of IRS1 at Ser302 is substantially induced by hyperinsulinemia [40], the lower insulin level (Table 1) may also contribute to the less phosphorylation of IRS1 at Ser302 in *Cpt1b*^+/−^ mouse muscle. The change in IRS1 phosphorylation could only be detected in skeletal muscle but not in other tissues, supporting that skeletal muscle is the main tissue responsible for the improved insulin sensitivity in *Cpt1b*^+/−^ mice under a HFD condition. Therefore, our results support that CPT1b repression sensitizes insulin action via increased glucose oxidation and insulin signaling in skeletal muscle. It is not clear why the insulin signal transduction in GWAT was suppressed at this point, but it appears that was not sufficient to offset the insulin sensitizing effect of partial CPT1b repression in skeletal muscle.

The insulin sensitizing effect of partial CPT1b deficiency appears to be unrelated to a decrease of relative body fat, since the fat mass was not correlated with mouse genotype assessed by ANCOVA. Despite the matched fat/lean mass ratio between the *Cpt1b*^+/−^ and WT mice, serum level of leptin was markedly higher in WT than in *Cpt1b*^+/−^ mice (Table 1). It is plausible that the greater total body fat in the HFD-fed WT mice may contribute to the higher serum level of leptin. It is intriguing that the *Cpt1b*^+/−^ mice showed a slower growth rate than their WT counterparts in response to HFD with proportional decreases of fat and lean mass. Albeit the exact mechanism underpinning this phenomenon is not clear, it is plausible that a potentially less energy production rate from glucose oxidation may play certain roles. Because of the lack of neurohumoral responses, the *ex vivo* oxidation assays using isolated muscle, isolated mitochondria, and homogenate may not reflect what exactly happens under the *in vivo* condition. This may explain why the CPT1b deficient mice showed repressed fatty acid oxidation and increased glucose oxidation in skeletal muscle, yet without major shift in whole body metabolism [42,43].

Interestingly, the effects of CPT1 deficiencies on growth and insulin resistance are subtype-dependent. One study found that *Cpt1c*^+/−^ mice are smaller than WT mice even under a CHD condition and they are susceptible to insulin resistance, obesity, and hepatosteatosis under a HFD condition [44]. Previously we reported that the *Cpt1a*^+/−^ mice were resistant against HFD-induced insulin resistance, but showed hepatosteatosis with increased hepatic CPT1b expression [45]. We also found that *Cpt1b*^+/−^ mice showed 40% decreased CPT1 activity in muscle and there were no significant changes in body weight, tissue histology, serum free fatty acid [16], and normal cardiac function and heart weight compared to WT littermates [46]. In this study we found that *Cpt1b*^+/−^ mice showed no difference in glucose and insulin tolerance under a CHD condition at least up to 32 weeks of age. Insulin sensitivity of *Cpt1b*^+/−^ mice is well maintained under a HFD condition. Therefore, it appears that CPT1b is a better specific CPT1 target for the treatment of insulin resistance.

Taken together, the current investigation confirmed the beneficial effects of CPT1b repression on diet-induced insulin resistance. Further studies will be required to identify CPT1b-specific inhibitors, and to confirm the effectiveness and safety of prolonged inhibition of CPT1b in animals and in patients with insulin resistance and type II diabetes.

## Supplementary Material

Supplementary File

## Figures and Tables

**Table 1:** Plasma lipid and hormone profile after 8 weeks, 22 weeks of HFD feeding, and insulin clamp (n=7–8).

Time	8 weeks of HFD		22 weeks of HFD		22 weeks of HFD Clamp	
strain	WT	CPT1b^+/−^	WT	CPT1b^+/−^	WT	CPT1b^+/−^
Glucose (mg/dl)	82.7 ± 3.3	73.1 ± 4*	148 ± 6.3‡‡	109 ± 11‡‡*	162 ± 28	158 ± 25
NEFA (mEq/L)	1.3 ± 0.2	1.0 ± 0.2	1.1 ± 0.1	1.3 ± 0.2	0.9 ± 0.14	0.5 ± 0.06*
Insulin (μIU/ml)	6.3 ± 0.4	6.3 ± 0.4	10.7 ± 0.9‡‡	8.3 ± 0.6‡*	37.5 ± 7.5	45 ± 4.9
Leptin (μg/L)	5.0 ± 2	27.1 ± 21	35.4 ± 11‡	19.4 ± 4.0	ND	ND

Mice were fasted for overnight. *n*=4–7,

* p<0.05,

** p<0.01 between WT and CPT1b^+/−^ mice within the same time point,

‡ p<0.05,

‡‡ p<0.01 between 8 and 22 weeks within the same group.

## Abbreviations

PKB: Protein Kinase B
CHD: Chow Diet
MAPK1: Mitogen-Activated Protein Kinase 1
FAO: Fatty Acid Oxidation
HFD: High Fat Diet
IRS1: Insulin Receptor Substrate 1
ITT: Insulin Tolerance Test
OGTT: Oral Glucose Tolerance Test
WT: Wild Type
