# Supplementary material for: Carnitine Palmitoyltransferase 1b Deficiency Protects Mice from Diet-Induced Insulin Resistance
Source: J Diabetes Metab. Author manuscript; Available in PMC 2014 Oct 8. (PMC4190034; doi:10.4172/2155-6156.1000361)

**Supplemental Table 1. DNA sequences of real-time qPCR primer sets.**

| Target Genes | Primer sequences |
|---|---|
| *Cpt1a* (carnitine palmitoyltransferase 1a) | F 5’-ACTCGCTGAAGGTGCTGCTCTC-3’ |
| | R 5’-GTGCTGTCATGCGTTGGAAGTC-3’ |
| *Cpt1b* (carnitine palmitoyltransferase 1b) | F 5’- TTCAACACTACACGCATCCC-3’ |
| | R 5’- GCCCTCATAGAGCCAGACC-3’ |
| *Cpt2* (carnitine palmitoyltransferase 2) | F 5’-GCTCCGAGGCATTTGTC-3’ |
| | R 5’-CATCGCTGCTTCTTTGGT-3’ |
| *Cd36* (fatty acid translocase) | F 5’-AAAGTTGCCATAATTGAGTCCT-3’ |
| | R 5’-TCCGAACACAGCGTAGATAGA-3’ |
| *Fatp* (fatty acid transport protein) | F 5’- CGGCGGTGGCGGAGGTGA-3’ |
| | R 5’-AGAAGCGGCTGGCGGAGAACT-3’ |
| *Fabp* (fatty acid binding protein) | F5’- TCGAGAAGAACGGGGATAC-3’ |
| | R 5’-CTGCCATGGGTGAGAGT-3’ |
| *Acs* (acyl-CoA synthetase) | F 5’- GCAAGAAGTGTGGGGTGGAAATCA-3’ |
| | R 5’-CATATGGGCGAGAGGCAAGAAAGA-3’ |
| *Ggat* (diacylglycerol O-acyltransferase 2) | F 5’- CGTGTGGCGCTACTTCCGAGACT-3’ |
| | R 5’- ACCAGCCAACGTAGCCAAATAGG-3’ |
| *Gpat* (glycerol-3-phosphate acyltransferase) | F 5’- GGAGTGTGGCGAGAGGCGTTATC-3’ |
| | R 5’- TTGCTGGCGGTGAAGAGAATGTG-3’ |
| *Mcd* (malonyl CoA decarboxylase) | F 5’- ATGAGGCTGTGTGCCTGGTA-3’ |
| | R 5’- TTGCTGTTGTTCTGGAACTGG-3’ |
| *Mcad* (medium chain acyl-CoA dehydrogenase) | F 5’-GTACCCGTTCCCTCTCATCAAA-3’ |
| | R 5’-CTCCGTCATCCTCCCCAAATAC-3’ |
| *Vlcad* (very long chain acyl-CoA dehydrogenase) | F 5’-TCGCTTGGCAGAGATTGTGG-3’ |
| | R 5’-AGGGGCTGGGTATGGCTGAG-3’ |
| *Glut4* (glucose transporter 4) | F 5’-TTCCAGCAGATCGGCTCTGA-3’ |
| | R 5’-AAGACATTGTTGGCCAGCAT-3’ |
| *Pparα* (peroxisome proliferator-activated receptor alpha) | F 5’- TGGACACAGAGAGCCCCAT-3’ |
| | R 5’- TGATGACAGAGCCCTCGGA-3’ |
| *Pparδ* (peroxisome proliferator-activated receptor delta) | F 5’-TCGGGCTTCCACTACGG-3’ |
| | R 5’- ACTGACACTTGTTGCGGTTCT-3’ |
| *β-actin* | F 5’- CTGTCCCTGTATGCCTCTG-3’ |
| | R 5’- GCGGTAGGGACAGTTCACAG-3’ |

**Supplemental Figure 1. *Cpt1b*$^{+/-}$ mice show no differences in glucose tolerance and insulin tolerance a CHD condition.** (A) OGTT in mice with 4 weeks of CHD. (B) ITT in mice with 4 weeks of CHD in mice with 32 weeks of CHD. (C) OGTT in mice with 32 weeks of CHD. (D) ITT. *n*=4-5.

**Supplemental Figure 2. Transcript analysis using RT-qPCR in gastrocnemius muscle.** The expression levels of Lipogenic genes are suppressed in *Cpt1b*+/- muscle compared to WT muscle. *n*=5-6, **p*<0.05, ***p*<0.01, ****p*<0.001.

Supplementary Figure 1.

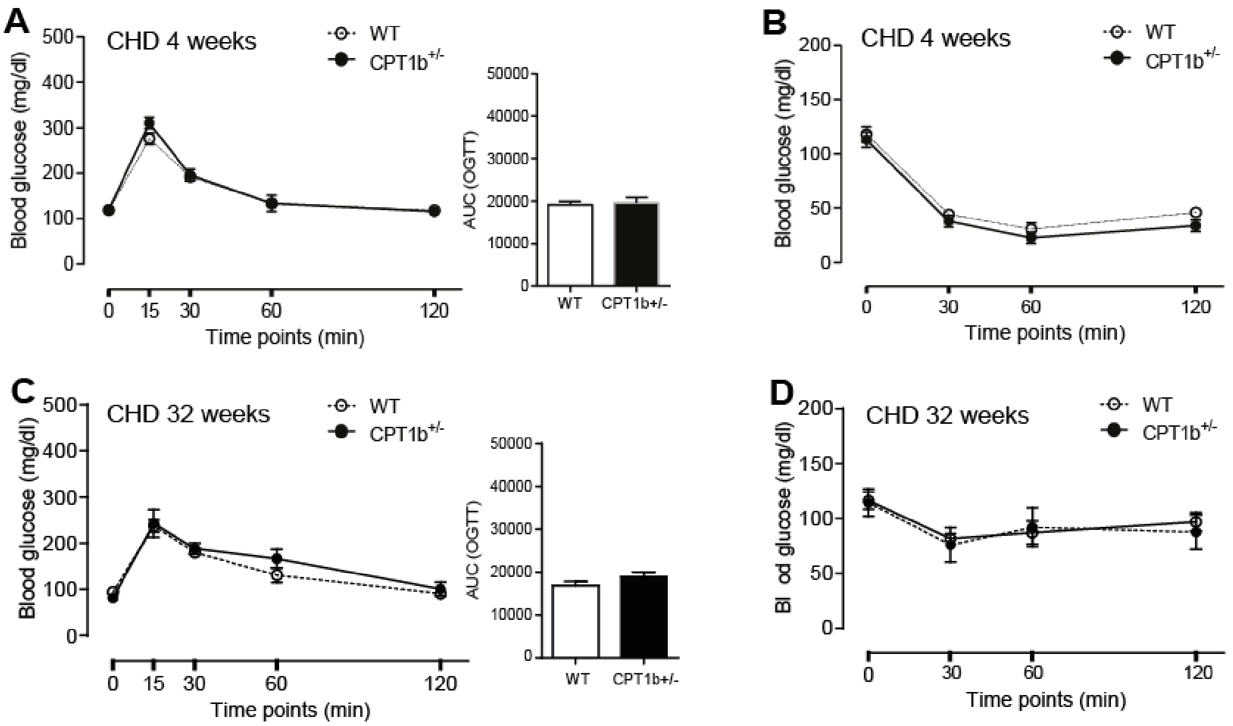

Supplementary Figure 2.

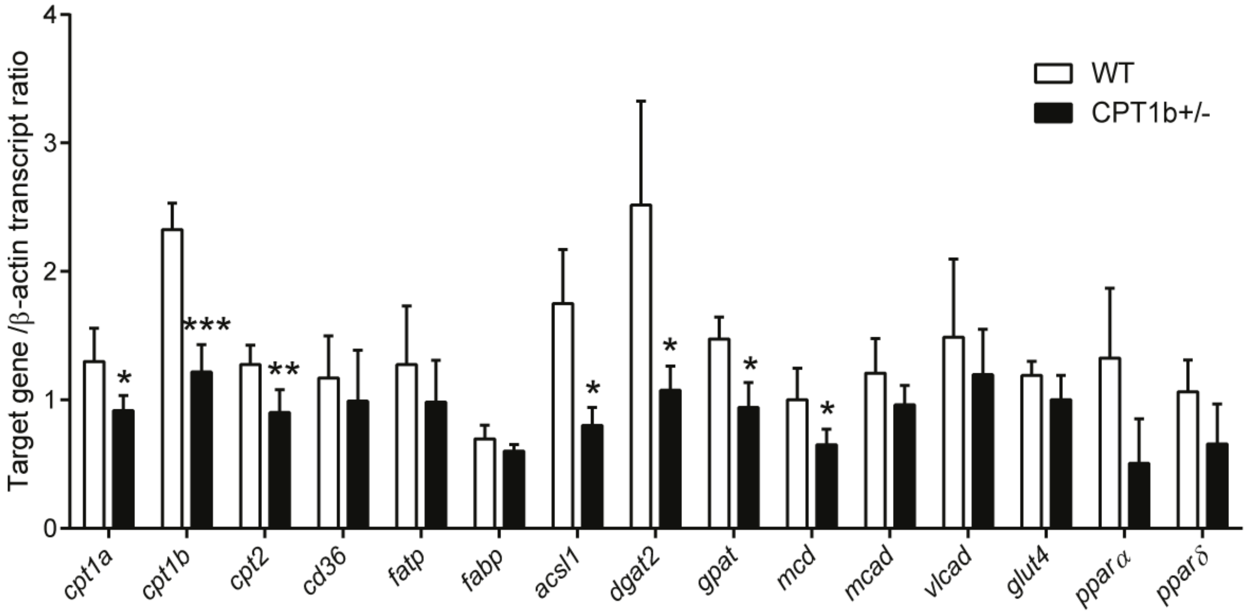

Supplement: Supplementary File [file NIHMS607072-supplement-Supplementary_File.pdf]
